# Comparing cardiovascular magnetic resonance strain software packages by their abilities to discriminate outcomes in patients with heart failure with preserved ejection fraction

**DOI:** 10.1186/s12968-021-00747-y

**Published:** 2021-05-20

**Authors:** Ying Zhang, David Mui, Julio A. Chirinos, Payman Zamani, Victor A. Ferrari, Yucheng Chen, Yuchi Han

**Affiliations:** 1 Cardiovascular Division, Department of Medicine, Perelman School of Medicine, University of Pennsylvania, 3400 Civic Center Blvd, Philadelphia, PA 19104 USA; 2 PLA General Hospital, Beijing, China; 3 Perelman School of Medicine, University of Pennsylvania, Philadelphia, PA USA; 4 Department of Cardiology, West China Hospital, Chengdu, China

**Keywords:** Feature tracking, Post-processing, CMR, HFpEF, Strain, Software package comparison

## Abstract

**Background:**

Cardiovascular magnetic resonance (CMR) myocardial strain analysis using feature tracking (FT) is an increasingly popular method to assess cardiac function. However, different software packages produce different strain values from the same images and there is little guidance regarding which software package would be the best to use. We explored a framework under which different software packages could be compared and used based on their abilities to differentiate disease from health and differentiate disease severity based on outcome.

**Method:**

To illustrate this concept, we compared 4-chamber left ventricular (LV) peak longitudinal strain (GLS) analyzed from retrospective electrocardiogram gated cine imaging performed on 1.5 T CMR scanners using three CMR post-processing software packages in their abilities to discriminate a group of 45 patients with heart failure with preserved ejection fraction (HFpEF) from 26 controls without cardiovascular disease and to discriminate disease severity based on outcomes. The three different post-processing software used were SuiteHeart, cvi42, and DRA-Trufistrain.

**Results:**

All three software packages were able to distinguish HFpEF patients from controls. 4-chamber peak GLS by SuiteHeart was shown to be a better discriminator of adverse outcomes in HFpEF patients than 4-chamber GLS derived from cvi42 or DRA-Trufistrain.

**Conclusion:**

We illustrated a framework to compare feature tracking GLS derived from different post-processing software packages. Publicly available imaging data sets with outcomes would be important to validate the growing number of CMR-FT software packages.

## Background

Cardiovascular magnetic resonance imaging (CMR) is recommended as the gold standard for the assessment of left ventricular (LV) systolic function [1]. Myocardial strain analysis using feature tracking (FT) has emerged to be a simple post-processing method to assess cardiac function in heart failure (HF) with preserved ejection fraction (HFpEF) patients [2, 3]. While HFpEF has previously been thought of as patients with diastolic dysfunction, new evidence has revealed that HFpEF patients also have systolic impairments [4–8]. HFpEF patients have reduced LV global circumferential strain (GCS) and global longitudinal strain (GLS) [5]. In fact, a reduced LV GLS has been shown to be an independent predictor for adverse cardiac outcomes in HFpEF patients [9].

The strain values produced by FT analysis is vendor-dependent. There are a number of different commercial software packages available for FT and different vendors produce different strain values [10]. There is no standardization or reference in the measurement of strain values by different vendors [11]. The approach generally taken by investigators to overcome these issues has been to establish normative values within each laboratory with each software package and use that local standard for all the analysis performed locally. Thus, which software package and strain analysis herein is able to better discriminate patients from non-cardiovascular disease controls and which software package can better discriminate patients with worse outcomes is not known.

In this study, we propose that different CMR FT software packages be compared and used based on their abilities to differentiate disease from health and to discriminate disease severity based on patient outcome. To illustrate this concept, we compared three CMR post-processing FT software packages SuiteHeart (Neosoft, Pewaukee, Wisconsin, USA), cvi42 (Circle Cardiovascular Imaging, Calgary, Alberta, Canada), and DRA-Trufistrain (Siemens Healthineers, Erlangen, Germany) in a group of HFpEF patients. We first compared their abilities to discriminate HFpEF patients from controls using GLS. In addition, we investigated if there was a difference in the GLS derived from different software packages to associate with outcomes in HFpEF patients.

## Methods

### Study design and population

We retrospectively analyzed baseline data from 45 HFpEF patients enrolled in a previous study [12] and 26 control patients who were referred for a clinical CMR study. Patients with HFpEF were included on the basis of symptomatic HF with LV ejection fraction (LVEF) > 50% and at least one of the following conditions: (1) prior hospitalization for decompensated HF; (2) intravenous diuretics or hemofiltration for short-term treatment of HF; (3) elevated filling pressures as measured by echocardiography; (4) long-term usage of loop diuretics; or (5) an elevated N-terminal pro-B-type natriuretic peptide (NT-proBNP). Subjects were all on stable medical therapy for the past month. Exclusion criteria included: (1) primary pulmonary vascular disease or significant pulmonary disease; (2) acute coronary syndrome or coronary revascularization within the past 60 days; (3) noncardiac conditions that significantly limited exercise; (4) any electrocardiogram (ECG) rhythm other than sinus with native conduction; (5) known diagnosis of hypertrophic, infiltrative or inflammatory cardiomyopathy; (6) pericardial disease; (7) clinically significant perfusion defects on stress imaging without subsequent revascularization; (8) significant valvular disease (i.e. moderate or greater aortic regurgitation or mitral stenosis); (9) uncontrolled hypertension (systolic blood pressure (BP) > 180 mm Hg or diastolic BP > 100 mm Hg); (10) prior reduced LVEF < 50%; (11) hemoglobin < 10 g/dL; and (12) current therapy with hydralazine or organic nitrates or elevations in liver function test results.

Control patients were retrospectively chosen from a prospective cohort study of patients referred for a CMR at the Philadelphia Veterans Affairs Hospital [13]. We selected patients with an LVEF > 60% and normal transmitral peak E/A ratio from echocardiography who did not meet any of the following exclusion criteria (1) cardiovascular disease including coronary artery disease, previous myocardial infarction, atrial fibrillation, atrial flutter; (2) pulmonary disease including asthma, chronic obstructive pulmonary disease; (3) obstructive sleep apnea; (4) transient ischemic attack; (5) peripheral vascular disease; and (6) patients on continuous positive airway pressure therapy.

All HFpEF patients and controls were followed prospectively for HF hospitalization and death until January 2019. The incidence of death or hospitalization for HF was ascertained via medical record review by trained physicians. Incident HF was identified by: (1) a discharge diagnosis of decompensated HF; (2) presence of new onset or worsening HF symptoms; (3) clinical or radiologic evidence of pulmonary congestion, invasive evidence of increased LV filling pressures or elevated BNP (> 300 ng/L or NT-proBNP > 1000 ng/L). The protocol was approved by the institutional review boards of the Philadelphia Veterans Affairs Hospital and the Hospital of the University of Pennsylvania. All subjects provided written informed consent.

### CMR acquisition

All participants had CMR examination on a 1.5 T CMR scanner (Avanto or Espree; Siemens Healthineers) using an eighteen-channel phased-array body coil. Retrospective ECG gated cine CMR imaging was performed with balanced steady-state free precession (bSSFP) sequences using standard protocol covering short axis and long axis in the 2- and 4-chamber views. The bSSFP cine images were acquired continuously from the mitral annulus to the apical level without gaps on the short axis. The LV outflow track (3-chamber) view was not consistently acquired and therefore not included in the analysis. Briefly, the imaging parameters are as follows: repetition time = 2.6 ms; echo time = 1.3 ms; phases = 30; slice thickness = 8 mm; bandwidth = 898 Hz/pixel; flip angle = 70°, field of view = 300 to 340 mm^2^; matrix size = 192 × 192; and parallel imaging factor = 2.

### CMR image analysis

Short axis cine images were used to analyze LV systolic function. The LV function analysis was performed using SuiteHeart 5.0.0 (Neosoft Inc) including all phases between end-diastole and end-systole of all slices. Papillary muscles were included in the ventricular volume and all volumetric parameters were indexed to body surface area. Longitudinal strains were analyzed on three different cardiac post-processing software packages SuiteHeart 5.0.0 (Neosoft Inc.), cvi42 5.10.3 (Circle Cardiovascular Imaging), and DRA-Trufistrain 2.1 (Siemens Healthineers). Long axis endocardial and epicardial contours of 2-chamber and 4-chamber cine images were automatically traced by SuiteHeart and cvi42 with manual correction. For DRA-Trufistrain, the endocardial and epicardial contours at end-diastole were manually traced and automatically propagated through the other phases based on the results of the deformable image registration. The peak global myocardial systolic strain (2-chamber and 4-chamber) was obtained for analysis. In order to delineate if contours play a role in the difference of the FT algorithms, we randomly selected 10 patients and compared the manually contoured results with the results from the automatic contours with manual correction.

### Statistical analysis

Continuous variables were described as mean ± standard deviation (SD) or median values based on normality of the variables. The normality of distribution of continuous variables was assessed by the Anderson–Darling test. Categorical variables were presented as percentages. The comparison of continuous parametric data was performed by Student’s t‐test and non-parametric data using Mann–Whitney test. Categorical variables were compared by Chi‐square test or Fisher’s exact test. The association of baseline clinical and imaging parameters and outcomes was evaluated by univariable Cox regression analysis and significant univariate parameters were to be adjusted in the multivariable analysis if appropriate. Receiver operating curves (ROCs) were used to determine optimal cut off values using area under the curve (AUC) and were compared using DeLong’s Test. The cut-off points were subsequently used in Kaplan–Meier curves to estimate the distribution of survival as a function of follow-up time. For all data, P < 0.05 was considered statistically significant. EmpowerStats (X&Ysolutions, China) was used to perform statistical analysis.

### Reproducibility analysis

We randomly selected 10 HFpEF patients to assess reproducibility. For inter-observer reproducibility, the second investigator completed the analysis independently. For intra-observer reproducibility, the same investigator analyzed the same group of subjects two months later blinded to the first measurements. Reproducibility was tested by intraclass correlation coefficient (ICC) and Bland–Altman plots.

## Results

### Clinical baseline characteristics

Forty-five HFpEF patients (64 ± 9 years, 75.6% male) and 26 non-HFpEF patients (58 ± 12 years, 80.8% male) were included in the analysis. The mean LVEF was lower in the HFpEF group (61.6 ± 7.0% vs 65.0 ± 6.4%, P = 0.039). During a median follow-up period of 3.8 years (range from: 1–5.2 years), 14 (31%) events occurred (9 HF hospitalizations and 5 deaths). All demographic and baseline clinical and CMR characteristics are shown in Table 1. At baseline, HFpEF patients demonstrated older age, higher body mass index (BMI), systolic BP, incidences of hypertension and obstructive sleep apnea.

**Table 1:** Baseline clinical and CMR characteristics of controls and HFpEF patients

Variable	Controls (N = 26)	HFpEF (N = 45)	P value
Age (years)	58.4 ± 12.3	64.3 ± 8.8	0.025
Male (n, %)	21 (80.8%)	34 (75.6%)	0.618
Race—White (n, %)	16 (61.5%)	15 (33.3%)	0.046
Race—African American (n, %)	10 (38.5%)	29 (64.4%)	0.034
BMI (kg/m^2^)	29.4 ± 5.6	36.5 ± 6.7	< 0.001
BMI > 30 (kg/m^2^)	10 (38.5%)	36 (80.0%)	< 0.001
*Cardiovascular related factors*			
Systolic blood pressure (mmHg)	139 ± 19	151 ± 20	0.016
Diastolic blood pressure (mmHg)	79 ± 13	87 ± 12	0.016
Hypertension (n, %)	16 (61.5%)	41 (91.1%)	0.002
Coronary artery disease (n, %)	4 (15.4%)	16 (35.6%)	0.070
Myocardial infarction (n, %)	1 (3.8%)	6 (13.3%)	0.296
Hyperlipidemia (n, %)	15 (57.7%)	38 (84.4%)	0.012
CVA or TIA (n, %)	2 (7.7%)	4 (8.9%)	0.864
Sleep apnea (n, %)	0 (0.0%)	22 (48.9%)	< 0.001
Smoker (n, %)	9 (34.6%)	8 (18.2%)	0.125
*CMR* *LV parameters*			
LVEDV index (ml/m^2^)	73.2 ± 13.1	75.1 ± 16.0	0.598
LVESV index (ml/m^2^)	25.6 ± 7.2	29.2 ± 9.6	0.100
LV SV index (ml/m^2^)	47.6 ± 8.6	45.9 ± 10.6	0.490
LVEF (%)	65.1 ± 6.4	61.6 ± 7.6	0.039

*CMR* cardiovascular magnetic resonance imaging, *HFpEF* heart failure with preserved ejection fraction, *BMI* body mass index, *CVA-TIA* cerebrovascular accident-transient ischemic attack, *OSA* obstructive sleep apnea, *LV* Left ventricle, *EDV* end diastolic volume, *ESV* end systolic volume, *SV* stroke volume, *EF* ejection fraction

### Automatic with manual correction versus manual contours

The mean difference and standard deviation of 4-chamber GLS determined from manual contours versus automatic contours with manual correction in 10 randomly selected patients using SuiteHeart is 0.21% and 0.87%, and using cvi42 is -0.19% and 0.61%, respectively. There was no statistical difference between SuiteHeart and cvi42 with p-values of 0.465 and 0.364, respectively. DRA-Trufistrain was not included in this comparison because it can only be manually traced.

### Discriminating HFpEF patients from controls

All global longitudinal strain parameters (2-chamber and 4-chamber) derived from the three software (SuiteHeart, cvi42, and DRA-Trufistrain) were able to distinguish HFpEF patients from non-HFpEF controls (p < 0.05) (Table 2).

**Table 2:** Discriminating HFpEF patients from controls using GLS

Software	Views	GLS strain	Control	HFpEF	P value
SuiteHeart	2C	Peak	− 19.4 ± 3.1	− 17.0 ± 3.5	0.012
		Mean	− 9.5 ± 2.10	− 8.3 ± 1.8	0.036
	4C	Peak	− 19.4 ± 3.7	− 17.1 ± 4.3	0.039
		Mean	− 9.4 ± 1.8	− 8.3 ± 2.1	0.039
cvi42	2C	Peak	− 16.9 ± 2.4	− 14.0 ± 3.2	< 0.001
		Mean	− 8.8 ± 1.6	− 7.3 ± 1.7	< 0.001
	4C	Peak	− 17.1 ± 3.0	− 13.1 ± 2.9	< 0.001
		Mean	− 8.7 ± 1.4	− 6.8 ± 2.0	< 0.001
DRA-Trufistrain	2C	Peak	− 14.6 ± 2.6	− 11.5 ± 3.1	< 0.001
		Mean	− 7.7 ± 1.5	− 6.1 ± 1.5	< 0.001
	4C	Peak	− 15.6 ± 3.0	− 11.3 ± 3.2	< 0.001
		Mean	− 7.9 ± 1.3	− 5.8 ± 1.6	< 0.001

*HFpEF* heart failure with preserved ejection fraction, *GLS* global longitudinal strain, *2C* 2-chamber, *4C* 4-chamber

### Discriminating outcomes of HFpEF patients using GLS

Strain parameters from SuiteHeart, cvi42, and DRA-Trufistrain were analyzed with regard to outcomes. Univariate logistic regressions were performed on baseline variables from Table 1 and none was found to be significantly associated with outcomes in HFpEF patients. 2-chamber peak GLS by all three software was not associated with outcomes in HFpEF patients. 4-chamber peak GLS by SuiteHeart was significantly associated with patient outcomes (HR = 1.2, 95% CI 1.0–1.4, p = 0.012) as compared to CVI^42^ (HR = 1.1, 95% CI 1.0–1.4, p = 0.130) and DRA-Trufistrain (HR = 1.2, 95% CI 1.0–1.5, p = 0.062) (Table 3).

**Table 3:** Association with outcomes (death or heart failure hospitalization) using 4-chamber GLS

Software	View	GLS Strain	Adjusted HR for adverse events	95% Confidence Interval	P value
SuiteHeart	4C	Longitudinal Peak	1.2	1.0 – 1.4	0.012
cvi42	4C	Longitudinal Peak	1.1	1.0 – 1.4	0.130
DRA-Trufistrain	4C	Longitudinal Peak	1.2	1.0 – 1.5	0.062

*GLS* global longitudinal strain; HR hazard ratio; *4C* 4-chamber

### Survival probability with outcomes using GLS

**Fig. 1:**
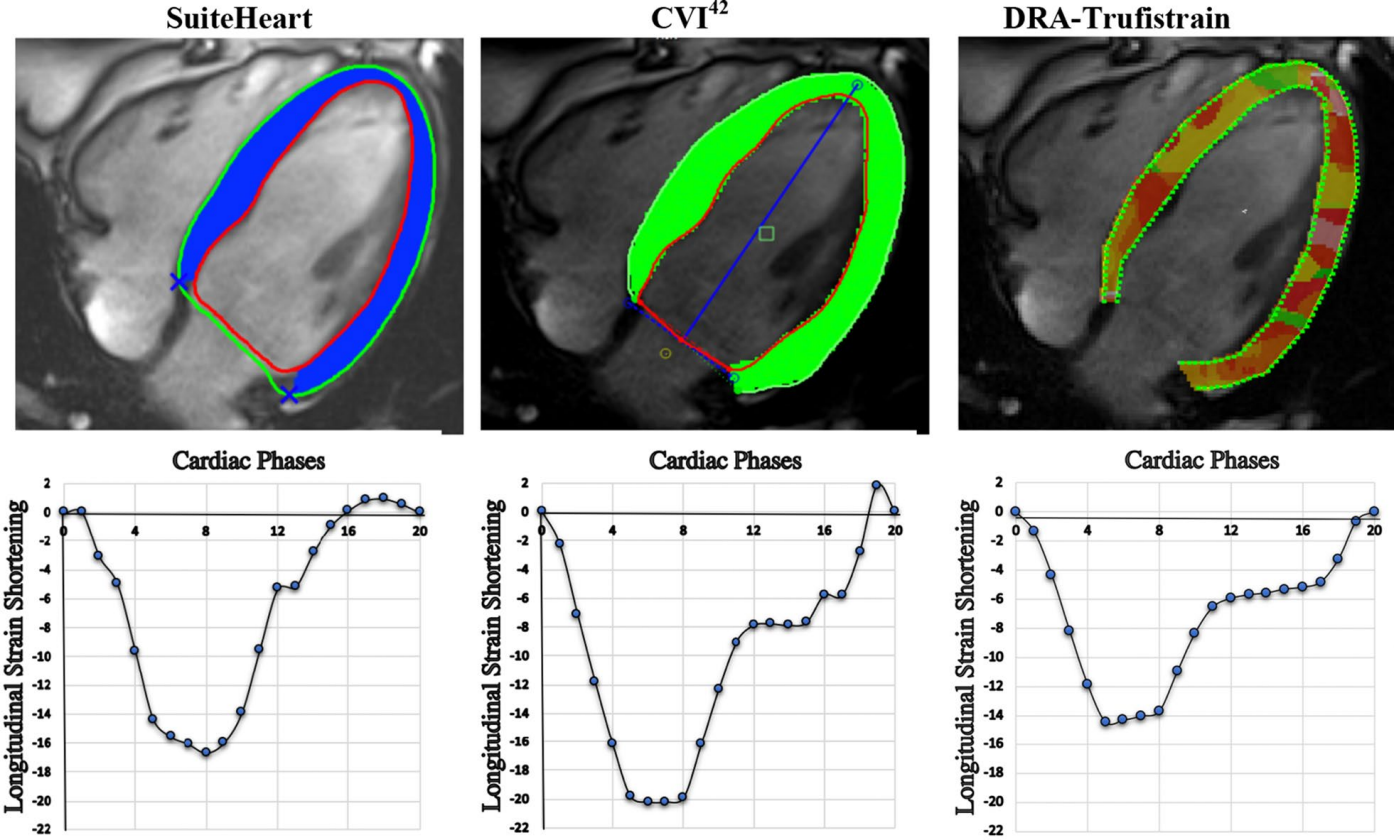
Images for longitudinal strain analysis from three post-processing software packages. The top panel shows examples of images by SuiteHeart, cvi42, and DRA-Trufistrain. In SuiteHeart, the endocardial and epicardial borders are marked by red and green contours, respectively, with myocardium marked in blue. In cvi42, the endocardial and epicardial borders are marked by red and green contours, respectively, with myocardium marked in green. In DRA-Trufistrain, the endocardial and epicardial borders are both marked by green contours . The bottom panel shows examples of longitudinal strain curves from SuiteHeart, cvi42, and DRA-Trufistrain. Different software packages produced different longitudinal strain values in the same patient

**Fig. 2:**
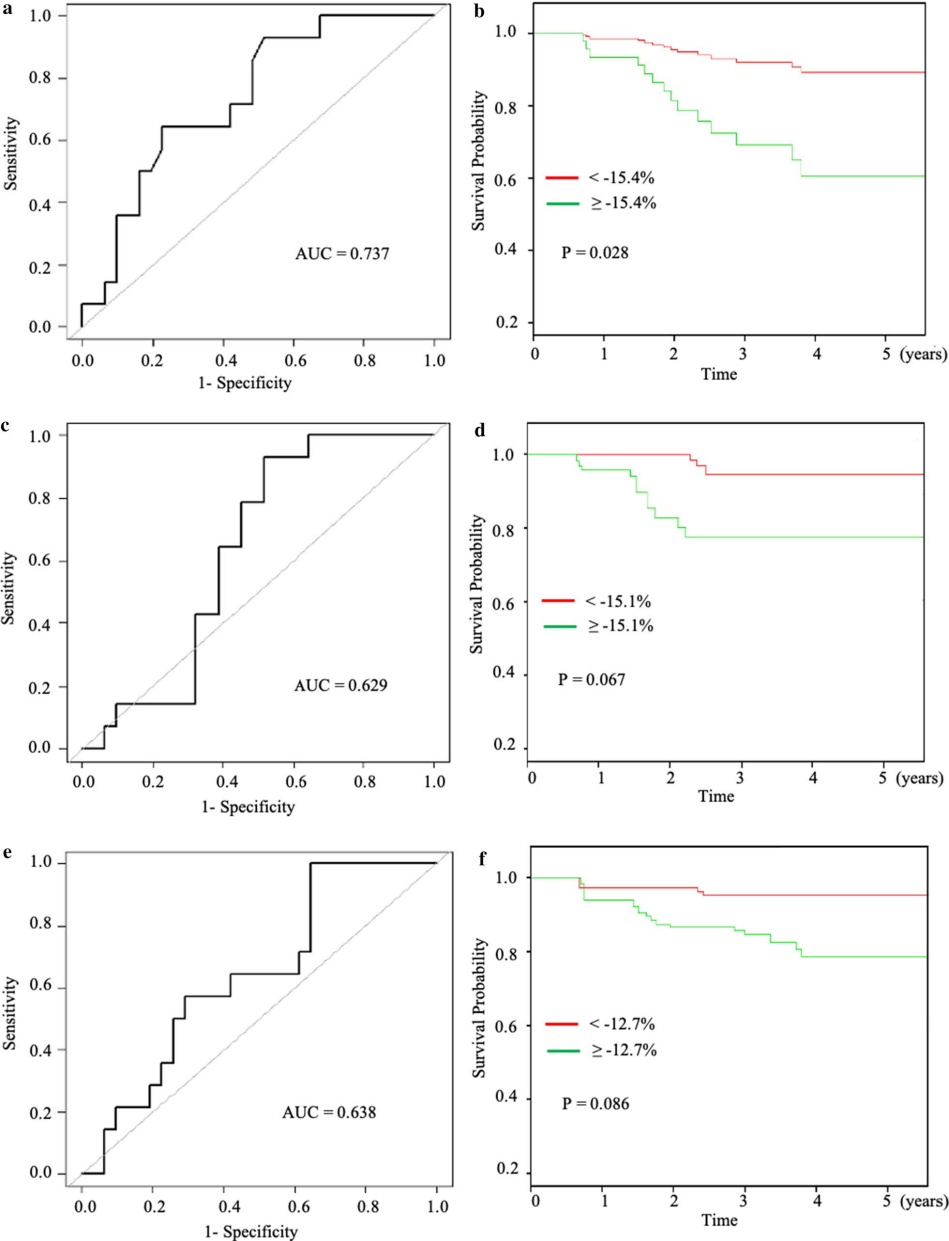
Receiver operating characteristics (ROC) curves and Kaplan Meier survival curves of HFpEF patients using the three software packages. The area under the curve (AUC) in ROC analysis (**a**, **c**, **e**) and Kaplan Meier curves (**b**, **d**, **f**) of death or heart failure hospitalizations using cut-offs derived from AUC analyses. **a** and **b** are for SuiteHeart, **c** and **d** are for cvi42, and **e** and **f** are for DRA-Trufistrain

ROC curves for SuiteHeart (AUC = 0.737, 95% confidence intervals [CIs] 0.590–0.860, optimal cut point = -15.4%; sensitivity = 0.64, specificity = 0.77), cvi42 (AUC = 0.629, 95% confidence intervals (CI) 0.470–0.770, optimal cut point = -15.1%; sensitivity = 0.93, specificity = 0.48), and DRA-Trufistrain (AUC = 0.638, 95% confidence intervals [CIs] 0.510–0.800, optimal cut point = -12.7%; sensitivity = 1.0, specificity = 0.35) were compared with De-Long’s test. Based on pairwise comparison of ROC curves, there was no significant difference in AUC between cvi42 and SuiteHeart (p = 0.225), cvi42 and DRA-Trufistrain (p = 0.599) and SuiteHeart and DRA-Trufistrain (p = 0.470). Using the optimal cut points determined from the ROC analysis, Kaplan Meier survival curves for the 3 software packages showed only SuiteHeart software was able to significantly differentiate the survival probabilities of the HFpEF patients (Figs. 1, 2).

### Reproducibility

**Fig. 3:**
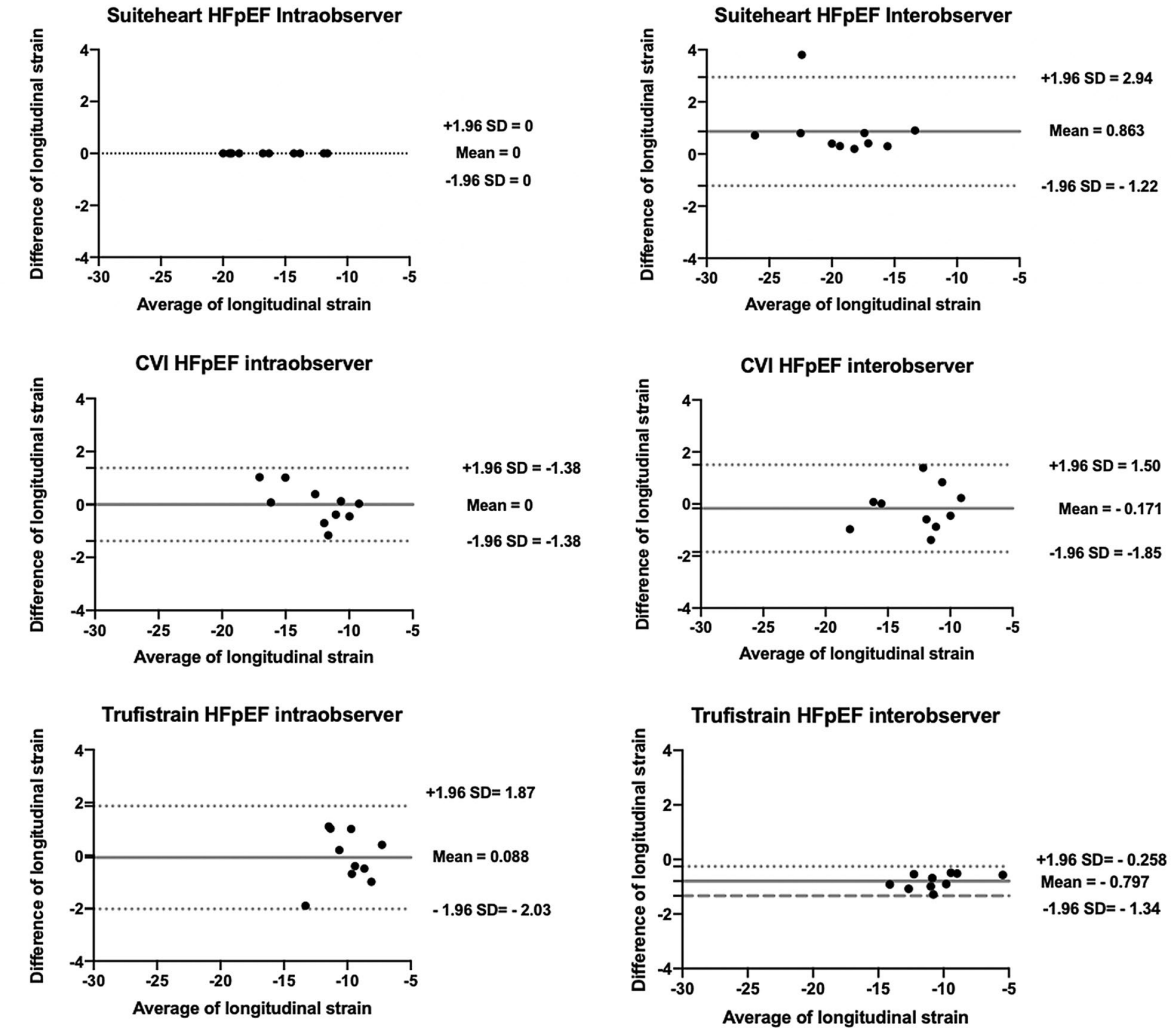
Intraobserver and interobserver variabilities as shown in Bland–Altman plots

All three software packages exhibited excellent intra and inter-observer reproducibility with the ICC ranging from 0.86 to 1.0 (SuiteHeart ICC 0.96 – 1.00, cvi42 ICC 0.86 – 0.99, DRA-Trufistrain ICC 0.88 – 1.00) on 2-chamber and 4-chamber longitudinal strains. Bland–Altman plots for inter- and intra-observer reproducibility for 4-chamber longitudinal strains in HFpEF patients are shown in Fig. 3.

## Discussion

We illustrated a framework by which CMR FT postprocessing strain software packages can be compared by evaluating its ability to discriminate HFpEF from non-HFpEF controls and further differentiate patients with adverse outcome. For the three software packages examined, all were able to discriminate HFpEF patients from controls. SuiteHeart 4-chamber GLS was associated with adverse outcomes of HFpEF patients in this small cohort of patients. cvi42 and DRA-Trufistrain were limited by the small sample size, but showed trends toward discrimination for adverse outcomes. The intra-observer and inter-observer reproducibilities in all three software packages were excellent. These findings may have significant implications for the clinical usage of different software packages in myocardial strain analyses.

Myocardial strain imaging techniques and analysis provide a valuable diagnostic and prognostic tool for assessing cardiac function [14]. CMR myocardial tagging (MT) serves as the gold standard technique for measuring myocardial strain and validating other strain measurement techniques [3, 15]. CMR MT utilizes spatial modulation of magnetization to create tags that move with the myocardium. This grid of myocardial tags can then be used to track cardiac deformation and measure strain [16]. Additional methods such as displacement encoding with stimulated echoes (DENSE) sequence can also be used to assess strain [17]. However, while CMR MT and DENSE are possibly the more accurate non-invasive techniques for measuring strain, they require acquisition of specialized images and complex post-processing, limiting their wide-spread use in clinical practice [11, 16].

CMR FT, on the other hand, is based on pattern-matching techniques of tracking “features” across multiple images in a cardiac cycle [16]. A pixel is identified in one frame and followed in the next successive frames, leading to tracking of myocardial deformations [18]. Different software packages use different often proprietary algorithms to perform tracking and thus result in different numerical values. These numerical values provided by CMR FT are also different from those derived from the CMR MT or DENSE and may not be as sensitive in disease detection [19]. Although there has been relatively good levels of agreement between FT and MT for globally measured strain, some FT software packages have been shown to consistently and systemically overestimate strain values [20]. Furthermore, intra- and inter-observer agreement of segmental strain by FT is lower than MT [21]. The fact that there are differences between the values produced by different FT software packages and from strains analyzed from tagging and DENSE is well known [22]. However, because FT does not require additional image acquisition and can estimate regional deformation using clinical bSSFP cine images, we will continue to see the growing use of FT in research and clinical settings. Thus, understanding the performance of different software packages and choosing appropriate software packages become important issues.

In addition to cvi42 and SuiteHeart, we included DRA—Trufistrain in our comparison. DRA stands for deformable registration algorithms. DRA also measures strain values from bSSFP cine images, but unlike most of the FT software which utilize optical flow methods and track endocardial features, DRA-Trufistrain method tracks the myocardium and produces layer-specific information [23]. DRA has been found to provide a reliable measurement of segmental and peak systolic strains with better accuracy and reproducibility than FT [23, 24].

In addition to CMR, 2D speckle-tracking echocardiography (STE) has also been widely used to assess myocardial strain due to its ease of use and availability. STE has been shown to have good correlation with the strain calculated by CMR FT and MT, although the agreement is not optimal in myocardial deformation analysis [25]. STE relies heavily on image quality and acoustic window for strain analysis, which can be challenging to consistently acquire [26]. There is currently no standardization in the calculation of myocardial strain in STE, leading to inter-vendor differences [14, 27]. Two vendors (General Electric Healthcare, Chicago, Illinois, USA, and Phillips Healthcare, Best, the Netherlands) and vendor-independent TomTec (TomTec Imaging Systems, Munich, Germany) all produce different strain values. Significant inter-vendor variability for 2D GLS measurements have led the European Association of Cardiovascular Imaging (EAVCI) and the American Society of Echocardiography (ASE) to set up a task force to assess the source of STE measurement variability in partnership with industry vendors [28]. Although inter-vendor agreements for STE have improved over time, the variability remains problematic when GLS is being used clinically across different vendor platforms.

Like echocardiography, strain values differ by vendor packages in CMR-FT. Worse than the problem in STE, where three main vendors differ and TomTec is the only vendor independent software, more than 10 vendor independent CMR FT software packages have emerged, which has amplified the problem of FT analysis in CMR. Each software utilizes different techniques to derive their strain measurements and because some methodologies are proprietary, it is difficult to directly compare these software methodologies. Furthermore, the selective use of different post-processing software may affect the significance of strain measurements. Although CMR is the gold standard for strain analysis using MT or DENSE, one study has found that 2D-STE provided stronger prognostic value to predict overall and CV mortality in HFpEF patients compared to CMR methods [29]. This discrepancy in the prognostic strain value obtained from CMR may be due to the specific post-processing software used. In our study, manually contoured 4-chamber longitudinal strain measurements from both SuiteHeart and cvi42 did not differ significantly from automatically contoured strain measurements. Therefore, we suspect that the performance differences among these two vendors is based on proprietary tracking algorithms, rather than the difference in contour segmentation. This underscores the need for standardization of CMR software for strain measurement [30]. One possible future solution is to make large datasets with outcomes available for software vendors to benchmark their algorithms.

Our study addresses one important concept in evaluating diagnostic and prognostic parameters or methodologies by directly examining the software package’s ability to detect and differentiate disease. Since it would be impossible to standardize individual algorithms, we propose to standardize methodology to evaluate output parameters from the different software packages. The goal is to find parameters from the analysis to differentiate disease from control and to detect more severe disease with worse prognosis from the less severe. In this particular study cohort, we have found that all three software packages investigated were able to fulfill the first requirement of differentiating disease from control, but only one was able to differentiate the more severe from the less severe disease in this small cohort of patients as evidenced by adverse outcome events. Both cvi42 and Trufistrain will likely be able to inform outcome if the sample size were larger, as evidenced by the p-value trends (Table 4). In fact, cvi42 had been shown  to predict outcomes in a larger cohort of patients [31].

**Table 4:** 4-chamber GLS receiver operating curve and Kaplan Meier curve analysis based on area under the curve derived cut-off values and follow-up time for death or heart failure hospitalization in HFpEF patients.

	AUC	Cut-off value	Specificity	Sensitivity	KM P-value
SuiteHeart	0.737	-15.4	0.77	0.64	0.028
cvi42	0.629	-15.1	0.48	0.93	0.067
DRA-Trufistrain	0.638	-12.7	0.36	0.63	0.086

*GLS* global longitudinal strain, *AUC* area under the curve, *KM* Kaplan Meier, *HFpEF* heart failure with preserved ejection fraction

### Limitations

Our study has a number of limitations. We only studied one disease to illustrate the concept. Our myocardial strain analysis also only focused on two parameters, GLS from 2-chamber and 4-chamber views. 3-chamber views were not used due to inconsistencies in slice selection in acquired images. We choose not to compare GCS on the short-axis view because when we compared GLS and GCS between HFpEF patients and controls, we found that the GLS had greater sensitivity for separation of patients and controls and thus chose to use GLS as an illustrative example. We did not perform global radial strain analysis due to its inferior reproducibility compared to GLS and GCS. Our goal is to provide a demonstration of the method of comparison of strain software using HFpEF as an illustrative example, rather than making a firm statement about strain and outcomes in HFpEF patients. Another limitation to our study is that we only included a single version of each of three software packages. We considered that these software packages to be representative to illustrate the concept as both SuiteHeart and cvi42 have artificial intelligence assisted contour tracking algorithms and are currently widely used. We also included DRA-Trufistrain as it represents a different tracking algorithm.

## Conclusions

In the setting of lacking standardization and increasing use of CMR FT in research and clinical realms, we proposed and demonstrated that the output of the CMR strain parameters can be evaluated across packages for disease discrimination and for adverse outcome differentiation. The most successful software package will be the ones that could perform well in different disease categories. Publicly available large data sets with outcomes in different disease populations by which the software developers could use to benchmark their products are urgently needed.

## Data Availability

Available upon request.

## Abbreviations

2D: 2 Dimensional
ASE: American Society of Echocardiography
BMI: Body mass index
BP: Blood pressure
bSSFP: Balanced steady-state free precession
CMR: Cardiovascular magnetic resonance
DENSE: Displacement encoding with simulated echoes
DRA: Deformable registration algorithms
EACVI: European Association of Cardiovascular Imaging
ECG: Electrocardiogram
FT: Feature tracking
GCS: Global circumferential strain
GLS: Global longitudinal strain
HF: Heart failure
HFpEF: Heart failure with preserved ejection fraction
ICC: Intraclass correlation coefficient
LV: Left ventricle/left ventricular
LVEDV: Left ventricular end-diastolic volume
LVEF: Left ventricular ejection fraction
LVESV: Left ventricular end-systolic volume
MT: Myocardial tagging
NT-proBNP: N terminal pro-B-type natriuretic peptide
STE: Speckle tracking echocardiography
SV: Stroke volume
